# Chlorophyll content and fluorescence as physiological parameters for monitoring *Orobanche foetida* Poir. infection in faba bean

**DOI:** 10.1371/journal.pone.0241527

**Published:** 2021-05-25

**Authors:** Moez Amri, Zouhaier Abbes, Imen Trabelsi, Michel Edmond Ghanem, Rachid Mentag, Mohamed Kharrat

**Affiliations:** 1 African Integrated Plant and Soil Research Group (AiPlaS), AgroBioSciences (AgBS), University Mohammed VI Polytechnic (UM6P), Ben Guerir, Morocco; 2 Carthage University, Field Crop Laboratory, National Institute for Agricultural Research of Tunisia (INRAT), Tunis, Tunisia; 3 Carthage University, Laboratory of Agronomic Sciences and Techniques, National Institute for Agricultural Research of Tunisia (INRAT), Tunis, Tunisia; 4 Biotechnology Unit, CRRA-Rabat, Regional Center of Agricultural Research of (INRA), Rabat, Morocco; Higher Institute of Applied Sciences and Technology of Gabes University of Gabes, TUNISIA

## Abstract

*Orobanche* spp. are root parasitic plants that cause yield losses in faba bean (*Vicia faba* L.). In Tunisia, *O*. *crenata* and *O*. *foetida* are among the major problems limiting faba bean production and productivity. Breeding for resistance and development of resistant varieties remain the most efficient control strategy to combat these parasites. In our study, field trials were conducted over two consecutive cropping seasons. A set of 42 genotypes were used in this study; 39 advanced lines and three checks; Najeh and Baraca (resistant) and Badi (susceptible). The trials were conducted in highly infested *O*. *foetida* plot at Oued-Beja Research Station in Tunisia. Results showed that advanced lines XAR-VF00.13-1-2-1-2-1 and XBJ90.04-2-3-1-1-1-2A expressed high resistance level exceeding those recorded for resistance checks Najeh and Baraca. *O*. *foetida* significantly affected the biomass, grain yield, chlorophyll content index (CCI) and the maximum quantum efficiency (F_v_/F_m_ ratio). No significant effect was observed on host plant water content (WC). CCI decreases varied from 46.4% for the susceptible check Badi and 4.2% and 9.3%, respectively, for Baraca and XBJ90.04-2-3-1-1-1-2A. *Orobanche* parasitism resulted in a slight decreases of F_v_/F_m_ ratio for the advanced lines XBJ90.04-2-3-1-1-1-2A and XAR-VF00.13-1-2-1-2-1 against important decreases observed for Badi and Baraca. Correlation between resistance to *O*. *foetida* and CCI and F_v_/F_m_ makes this, easy-to-measure, parameter very useful as a practical screening tool for early parasitism detection, diagnosis and identification and selection of high resistant plants against this parasite.

## Introduction

Broomrapes (*Orobanche* spp.) are holoparasitic plants completely dependent on the host for their nutritional requirements. In the Mediterranean region, where broomrapes are considered as a serious threat, they cause important damages and yield losses on many legume crops [1–3]. In Tunisia, *O*. *foetida*, *O*. *crenata*, *O*. *cumana*, and *Phelipanche ramosa* are parasitizing many crops such as faba bean, chickpea, lentil, grass pea and sunflower [4, 5]. While *O*. *crenata* was mentioned as a serious pest for decades, *O*. *foetida* has been presented as an emerging problem for many legume crops such as faba bean, chickpea, lentil, grass pea, medick, common and narbon vetch [4, 6, 7]. The *Orobanche* infested area in Tunisia is estimated to more than 80,000 ha mostly situated in the main grain legumes production area (unpublished data, authors’ own estimates). In high infested fields, farmers abandoned planting legumes especially faba bean and switched to non-host crops such as wheat leading to a strict wheat mono-cropping system. The devastating effect of *Orobanche* is associated with their multiplication rate and high seed viability (15–20 years) [8]. Many control methods were used to control *Orobanche*, including agricultural practices, chemical and biological methods [9, 11, 14]. The chemical method using especially synthetic strigolactones and herbicides were the most explored but resulted in a limited success due to their application complexity in the farmer’s field [8]. Till date, no single control method has shown successful with full control of the parasite. All control strategies resulted in an incomplete protection of the crop [9–11]. To date, the only effective method is through an integrated management strategy with genetic resistance as a main component. Farmers should use resistant varieties, avoid planting contaminated seeds and follow preventive practices to limit the spread of the parasite to new fields [12]. While avoidance of broomrape dispersal is virtually difficult, crop resistance and prevention measures could be the most effective and economical methods to reduce this root parasitic weed infestations. Genetic resistance coupled with other control methods resulted someway in good control of the parasite with significant decreases of the damages. At this level, research is needed for generating new technologies and developing new resistant varieties and effective screening tools. Many resistance mechanisms were studied focusing mainly on the physical and biochemical host-parasite interface such as low production of *Orobanche* seed germination stimulants and/or release of inhibitors by the host root system [2, 13, 14], the existence of a host plant roots physical barrier resulting in unsuccessful haustorial penetration and necrosis [15] and the development of a deep root system that escape *Orobanche* infestation [16, 17]. In addition, an integrated control strategy could be improved through early detection and monitoring of the underground infestation and the parasite development. Rousseau et al. [18], mentioned that plant infestation by root parasitic weeds has a systemic impact that could be observed on host leaves. In this regard, chlorophyll fluorescence, which is a non-destructive and rapid assessing tool of photochemical quantum yield and photoinhibition, could be used for early *Orobanche* infestation and estimate its impact on the host plant. It is widely used as a plant response indicator under biotic and abiotic constraints such as heat, drought, waterlogging, salt stress, nitrogen deficiency, pathogen infection and herbicide resistance [19–21]. However, only few studies were conducted on parasitism effect on host plant chlorophyll fluorescence [22–24]. As reported by Maxwell and Johnson [25], the photochemical processes alterations are usually the first signs in the stressed plant leaves that could be used to estimate photosynthetic performance under stress conditions. These photochemical processes alterations appear in the chlorophyll fluorescence kinetics and induce changes in the established fluorescence parameters and consequently PSII damages. Cameron et al. [26], reported that the parasitic plant *Rhinanthus minor* significantly reduced biomass production in *Phleum bertolinii* and demonstrated that such decrease was reflected by changes in photosynthetic activities and significant reductions in the quantum efficiency of PSII and chlorophyll concentration.

In this paper we aim to evaluate the performances of faba bean advanced lines collection under *O*. *foetida* infested conditions and assess the impact of the parasite on plant growth and seed yield in correlation with physiological behavior using chlorophyll content and chlorophyll fluorescence parameters.

## Material and methods

### Plant material and field trials

#### Genotypes evaluation and screening for resistance to *O*. *foetida*

A set of 39 small-seeded faba bean advanced lines, developed from crosses performed in Tunisia (Table 1), were used for a first-year (2009/2010) screening and evaluation for resistance to *O*. *foetida*. Three checks were added to the list, two Tunisian varieties Badi and Najeh and a Spanish variety Baraca. Both varieties Najeh and Baraca, carrying partial resistance to *O*. *foetida* and *O*. *crenata* [1, 27] were used as resistance check while Badi was used as susceptible check. The screening was performed under high *O*. *foetida* infested sick plot at Oued-Beja Research Station—Tunisia (36°44’N; 9°13’E). The trial was conducted in a randomized complete block design with three replications. Each genotype was planted, at a density of 24 seeds per m^2^, in four rows of 4 m length and 50 cm inter-rows spacing. The planting was performed the last week of November. No fertilizer’s supply or herbicide treatments were applied after plant emergence, only hand weeding was carried out keeping only faba bean plants and emerged *Orobanche* shoots.

**Table 1:** Origin and main characteristics of different studied genotypes.

Genotypes	Cross/Pedigree and main characteristics
XBJ90.04-6-2-1-1-4-C	Sel.88Lat.18035 x POL27-3
XBJ90.04-2-3-1-1-1-2A	Sel.88Lat.18035 x POL27-3
XBJ90.04-2-3-1-1-1	Sel.88Lat.18035 x POL27-3
XBJ90.03-20-3-1	Sel.88Lat.18025xSP49C
XBJ90.01-7-2-1-1-1-2-1-2	Sel.88Lat.18105 x POLTN-53-2
XBJ92-10-46-1-3-2-1-8-A	Sel.88Lat.15035 x (B8811 x LPF87)
XBJ92-10-46-1-3-1-2-1-1-1-6-A	Sel.88Lat.15035 x (B8811 x LPF87)
XBJ92.10-45-1-2-3-1-1	Sel.88Lat.15035 x (B8811 x LPF87)
XBJ92.10-27-1-2-1-1-1	Sel.88Lat.15035 x (B8811 x LPF87)
XBJ92.10-27-1-1-1-1-1	Sel.88Lat.15035 x (B8811 x LPF87)
XBJ92.10-17-1-3-2-1-1	Sel.88Lat.15035 x (B8811 x LPF87)
Syn1- (XBJ92-10-46-1-3)	A synthetic line: Sel.88Lat.15035 x (B8811 x LPF87)
XBJ93.12-10-1-1-3	S82-113-8 x Mateur
XAR-VF00.13-8-5-1-2-1	XBJ90.03-20-1-1-1-1-1-1-D (Sel.88Lat.18025x Giza402) x 19TB
XAR-VF00.13-8-3-2-2-2	XBJ90.03-20-1-1-1-1-1-1-D (Sel.88Lat.18025x Giza402) x 19TB
XAR-VF00.13-8-3-2-2-1	XBJ90.03-20-1-1-1-1-1-1-D (Sel.88Lat.18025x Giza402) x 19TB
XAR-VF00.13-8-3-1-2-1	XBJ90.03-20-1-1-1-1-1-1-D (Sel.88Lat.18025x Giza402) x 19TB
XAR-VF00.13-8-3-1-1-1	XBJ90.03-20-1-1-1-1-1-1-D (Sel.88Lat.18025x Giza402) x 19TB
XAR-VF00.13-8-1-2-1-2	XBJ90.03-20-1-1-1-1-1-1-D (Sel.88Lat.18025x Giza402) x 19TB
XAR-VF00.13-81-1-3-2-1	XBJ90.03-20-1-1-1-1-1-1-D (Sel.88Lat.18025x Giza402) x 19TB
XAR-VF00.13-81-1-3-1-1	XBJ90.03-20-1-1-1-1-1-1-D (Sel.88Lat.18025x Giza402) x 19TB
XAR-VF00.13-7-5-2-2	XBJ90.03-20-1-1-1-1-1-1-D (Sel.88Lat.18025x Giza402) x 19TB
XAR-VF00.13-70-1-1-1-1	XBJ90.03-20-1-1-1-1-1-1-D (Sel.88Lat.18025x Giza402) x 19TB
XAR-VF00.13-5-3-1-1-1	XBJ90.03-20-1-1-1-1-1-1-D (Sel.88Lat.18025x Giza402) x 19TB
XAR-VF00.13-4-5-1-1	XBJ90.03-20-1-1-1-1-1-1-D (Sel.88Lat.18025x Giza402) x 19TB
XAR-VF00.13-4-2-1-1	XBJ90.03-20-1-1-1-1-1-1-D (Sel.88Lat.18025x Giza402) x 19TB
XAR-VF00.13-31-7-2-1-1	XBJ90.03-20-1-1-1-1-1-1-D (Sel.88Lat.18025x Giza402) x 19TB
XAR-VF00.13-31-4-2-2-1	XBJ90.03-20-1-1-1-1-1-1-D (Sel.88Lat.18025x Giza402) x 19TB
XAR-VF00.13-27-5-1-2-1	XBJ90.03-20-1-1-1-1-1-1-D (Sel.88Lat.18025x Giza402) x 19TB
XAR-VF00.13-27-2-2-1-1	XBJ90.03-20-1-1-1-1-1-1-D (Sel.88Lat.18025x Giza402) x 19TB
XAR-VF00.13-27-2-1-1-4	XBJ90.03-20-1-1-1-1-1-1-D (Sel.88Lat.18025x Giza402) x 19TB
XAR-VF00.13-2-5-1-3-1	XBJ90.03-20-1-1-1-1-1-1-D (Sel.88Lat.18025x Giza402) x 19TB
XAR-VF00.13-24-2-2-1-2	XBJ90.03-20-1-1-1-1-1-1-D (Sel.88Lat.18025x Giza402) x 19TB
XAR-VF00.13-2-4-1-1-1	XBJ90.03-20-1-1-1-1-1-1-D (Sel.88Lat.18025x Giza402) x 19TB
XAR-VF00.13-2-2-2-3-2	XBJ90.03-20-1-1-1-1-1-1-D (Sel.88Lat.18025x Giza402) x 19TB
XAR-VF00.13-2-1-1-4-1	XBJ90.03-20-1-1-1-1-1-1-D (Sel.88Lat.18025x Giza402) x 19TB
XAR-VF00.13-2-1-1-1-1	XBJ90.03-20-1-1-1-1-1-1-D (Sel.88Lat.18025x Giza402) x 19TB
XAR-VF00.13-1-2-1-2-1	XBJ90.03-20-1-1-1-1-1-1-D (Sel.88Lat.18025x Giza402) x 19TB
Sel.88.Lat.18054-2-1-1	Originated from ICARDA
Badi	High yielding variety, released in Tunisia in 2004, susceptible to *O*. *foetida* and *O*. *crenata*.
Najeh	High yielding variety developed from the cross Sel.88Lat.18025xSP49C performed in Beja-Tunisia. Released and registered in Tunisia in 2009.
Baraca	High yielding variety released in Spain. Derived from the line VF1071 (a selection from F402 (Giza402)) as the original source of resistance to *O*. *crenata*.

#### Confirmation of the resistance and assessment of the parasitism impact

Out of the total tested collection, the two best resistant genotypes XAR-VF00.13-1-2-1-2-1 and XBJ90.04-2-3-1-1-1-2A were selected all with the three checks to conduct the second-year (2010/2011) evaluation and confirmation trials. These genotypes were selected not only based on the cropping season (2009/2010) but also from preliminary data and observations recorded during previous cropping seasons (data not shown). The five genotypes were planted the last week of November in both infested and free *Orobanche* fields. Both trials were conducted same as described in the first-year screening.

For both cropping seasons, monthly rainfall and average temperature distribution for the two cropping seasons collected from the iMETOS meteorological station (Pessl instruments) are presented in the Table 2.

**Table 2:** Climatic data (monthly minimum, maximum and average temperature (°C) and rain (mm) recorded in Beja research station during the two cropping seasons 2009/2010 and 2010/2011.

Cropping season	Temp./Rain	Sep	Oct	Nov	Dec	Jan	Feb	Mar	Apr	May	Jun	Avg	Total
2009–10	Temp. min	17.7	14.1	8.1	7.8	6.6	5.9	6.9	10.4	11.7	15.4	10.5	-
	Temp. max	30.3	24.7	21.5	18.5	15.8	17.6	19.7	22.9	26.7	32.1	23.1	-
	Rain (mm)	89.5	59.4	47.4	64.2	10.7	67.1	78	46.4	27.2	4.8	-	494.7
2010–11	Temp. min	17.3	13.6	10.7	6.2	5.6	5.1	6.4	9.5	12.3	15.2	10.2	-
	Temp. max	30.7	26.7	20.8	17	15.9	15.5	18.3	23.5	26.4	31.5	22.6	-
	Rain (mm)	42.7	82	56.8	61.6	63.2	138.4	58.3	38.6	43.4	7.4	-	592.4

### Measurements

The field evaluation of the studied genotypes and assessment of their resistance level to *O*. *foetida* was evaluated through different parameters measured at different crop development stages.

During the first-year screening, data related to Parasitism Index (PI), number of emerged *Orobanche* shoots (EOS) per faba bean plant and seed yield (SY) (g.m^-2^) were recorded at harvesting time on the two central rows.

PI=(OIN*OSV)/100

- OIN: *Orobanche* incidence or percentage of plants showing at least on *Orobanche* emerged shoot
- OSV: *Orobanche* severity (1–9 scale) or level of damage caused by *Orobanche* on the host plant development and seed production [28].

During the second-year evaluation, in addition to OIN, OSV, PI, EOS and SY mentioned above, the number of days to *Orobanche* emergence (NDOE) and total emerged *Orobanche* number per plant were also recorded. At pod-setting stage and from both infested and non-infested fields, five random faba bean plants from each plot were carefully dug-up with all *Orobanche* attachments. For each single plant the biomass and water content (WC) were recorded and *Orobanche* attachments (TON) were classified into emerged and non-emerged attachments.

Chlorophyll content index (CCI) and the maximum quantum efficiency (F_v_/F_m_ ratio) measurements were recorded once a week between 10 am and 1 pm from 123 to 144 days after planting (DAP) in both infested and non-infested fields. The two parameters were recorded on leaves from the 11^th^ main stem node of random faba bean plants. CCI was measured on five random faba bean plants per plot using an CL-01 Chlorophyll Content Meter (*Hansatech Instruments Ltd*, *UK)*. For every measurement almost the same part of the leaf was placed between two clips and the CCI was determined in dual wavelength optical absorbance (620 and 940 nm). F_v_/F_m_ ratio measurements were performed on two random faba bean plants from the two central rows using a Plant Efficiency Analyzer (*Handy-PEA*, *Hansatech instruments Ltd*, *P02*.*002 v*.). For each plant, almost the same part/point of the leaflet was delimited by measure clip and was maintained in dark during 16 min by closing the clip shutter. Dark adaptation time was required to obtain a steady state value of the ratio of variable to maximum fluorescence. After 16 min, chlorophyll fluorescence transients were induced by a red light of 1500 μmol.m^-2^.s^-1^ intensity.

Plant sampling, biomass, WC, CCI and the F_v_/F_m_ ratio measurements were performed only during second cropping season (2010/2011).

### Statistical analysis

The statistical analysis, ANOVA and means comparison, were performed using the SPSS statistical program v.21. Differences among treatments for all measurements were compared at *P* = 0.05 and using Duncan’s multiple-range test.

## Results

### Field evaluation and identification of potential resistance genotypes to *O*. *foetida*

**Fig 1:**
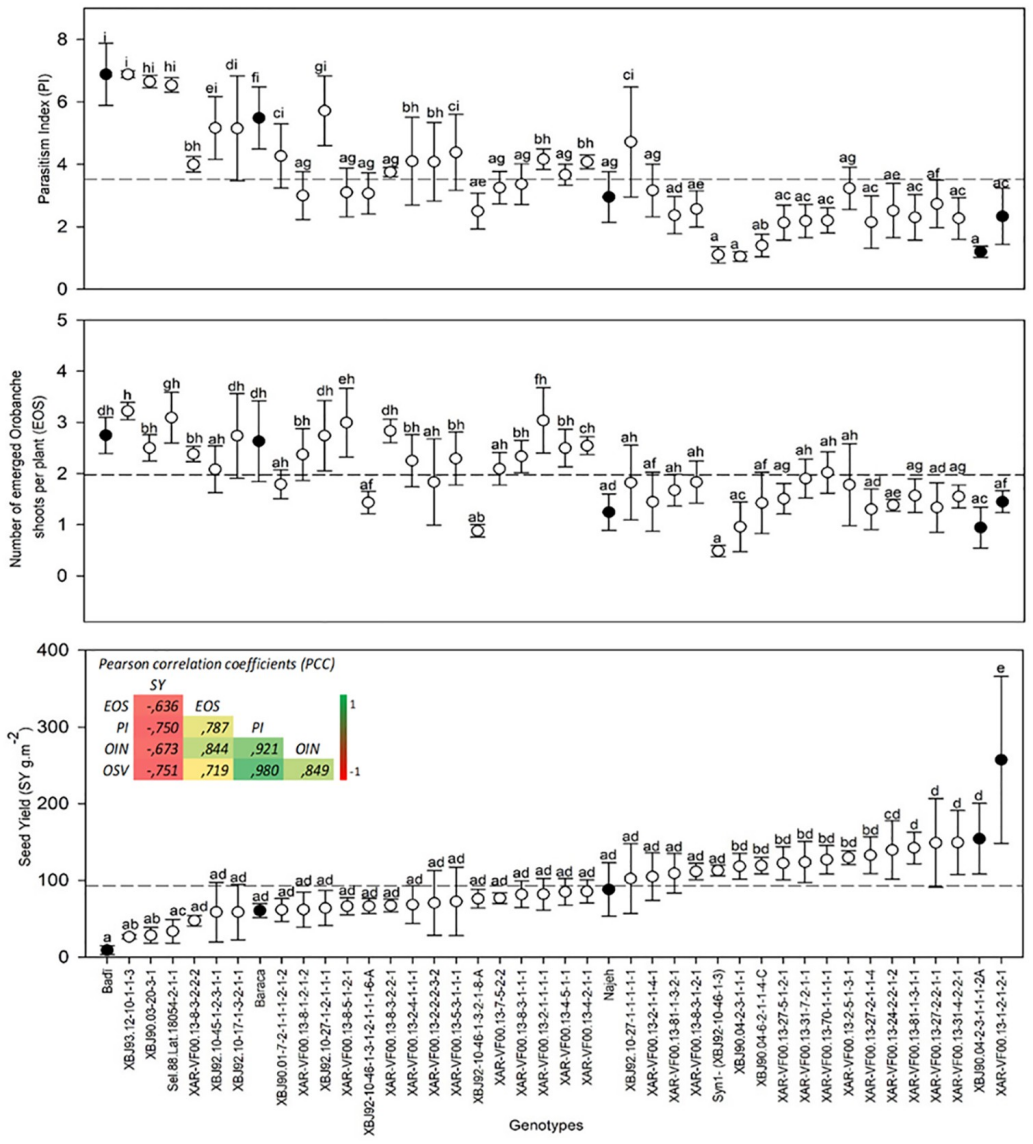


Results showed high variability in the resistance to *O*. *foetida* between the genotypes. Significant differences were observed for the EOS, PI and SY (Fig 1). Based on PI, almost 46% of the tested genotypes showed a resistance level higher than the resistant check Najeh. The two advanced lines XBJ90.04-2-3-1-1-1-2A and XAR-VF00.13-1-2-1-2-1 expressed a high resistance level to *O*. *foetida* with respective PI of 1.2 and 2.2. Both genotypes showed low *Orobanche* infestation level with only 0.9 and 1.4 EOS, respectively. Such resistance observed for these two genotypes was reflected by a high seed yield with 154.2 g.m^-2^ and 257 g.m^-2^ respectively. They produced almost two (1.8) and three (2.9) times more than the resistant check Najeh. High negative correlation between SY and both EOS (r = - 0.636, *P* ≤ 0.001) and PI (r = - 0.770, *P* ≤ 0.001) was observed.

### Confirmation of the resistance under infested and non-infested field conditions

**Fig 2:**
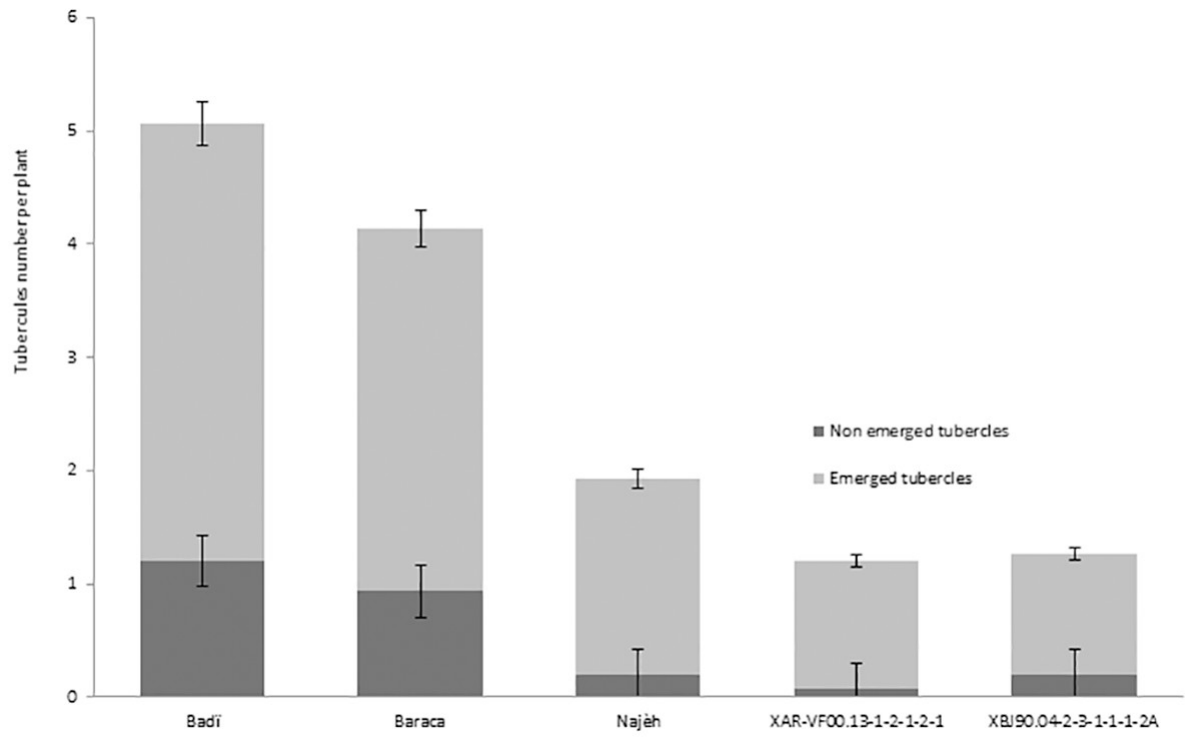


Results generated from the second-year evaluation showed high differences (*P* ≤ 0.01) between the five studied genotypes for OIN, OSV, DOE, TON, and EOS. High OIN was observed for the two cultivars Badi and Baraca with 81.7% and 85%, respectively (Table 3) against moderate to low OIN observed for Najeh (65%), XAR-VF00.13-1-2-1-2-1 (60%) and XBJ90.04-2-3-1-1-1-2A (40%). Maximum infestation was observed for the susceptible genotype Badi with 5 attachments per plant against only 1.2, 1.3 and 1.9 attachments observed for Najeh, XAR-VF00.13-1-2-1-2-1 and XBJ90.04-2-3-1-1-1-2A (Fig 2). At crop maturity (early June), EOS varied from 0.9 observed for XBJ90.04-2-3-1-1-1-2A to 2.7 recorded for Badi (Table 3). Only, 1.2, 1.4 and 2.6 shoots per plant were recorded respectively for Najeh, XAR-VF00.13-1-2-1-2-1 and Baraca. The NDOE varied from 133 days for the susceptible check cv. Badi to 145 days recorded for XBJ90.04-2-3-1-1-1-2A (Table 3). Compared to Badi, delay of 2.7, 4, 4.3 and 11.7 days were observed for NDOE for the genotypes XAR-VF00.13-1-2-1-2-1, Baraca, Najeh and XBJ90.04-2-3-1-1-1-2A, respectively. *Orobanche* severity varied from a minimum of 3 for XBJ90.04-2-3-1-1-1-2A to a maximum of 6.3 for Badi (Table 3). Such infestation levels resulted in a significant negative parasitism impact on plant growth and seed production for different tested genotypes.

**Table 3:** *Orobanche* incidence (%) and *Orobanche* severity (1–9), Number of Days to *Orobanche* Emergence (NDOE) and number of Emerged Orobanche Shoots per plant (EOS) recorded for different studied genotypes in high *O*. *foetida* infested field during the cropping season 2010/2011.

	*Orobanche* incidence (OIN) (%)	*Orobanche* severity (OSV) (1–9)	Number of days to *Orobanche* emergence (NDOE)	Emerged *Orobanche* shoot per plant at harvesting (EOS)
Badi	100.0±0^c^	6.3±1.2^b^	133.0±2.6^a^	2.7±0.6^c^
Baraca	76.7±25.2^bc^	4.3±1.2^a^	139.7±6.1^ab^	2.6±1.4^b^
Najeh	50.0±30^b^	3.0±0^a^	141.7±5^b^	1.2±0.6^a^
XAR-VF00.13-1-2-1-2-1	70.0±17.3^bc^	4.3±1.2^a^	140.3±3.1^bc^	1.4±0.4^a^
XBJ90.04-2-3-1-1-1-2A	13.3±5.8^a^	3±0.0^a^	145±2^a^	0.9±0.7^a^

NDOE: number of days from planting to the first *Orobanche* shoot emergence per plot. Values followed by the same letter column are not significantly different at p = 0.05 (Duncan test).

#### *Orobanche* parasitism effect on biomass and seed yield

Compared to non-infested field, results showed that *O*. *foetida* has significantly affected the host plant biomass (*P* ≤ 0.01) for all the genotypes (Table 4). A maximum decrease of biomass production (68.5%) was observed for the susceptible check Badi against only 15.6% recorded for Najeh. Respective decreases of 31%, 22.5% and 21.2% were recorded for Baraca, XAR-VF00.13-1-2-1-2-1 and XBJ90.04-2-3-1-1-1-2A (Table 4). For all tested genotypes, *Orobanche* parasitism has significantly affected the host plant biomass production but with no significant effect on the water content. No decreases in WC were observed between infected and non-infected plants (Table 4).

**Table 4:** Biomass and water content (WC) recorded for different studied genotypes in both *O*. *foetida* infested and non-infested fields.

	Non-infested field		*O*. *foetida* infested field	
	Biomass	WC	Biomass	WC
Badi	313.4±122.9^b^	76,8±4,9^bc^	98.7±67.4^a^	70,3±16,3^a^
Baraca	220.3±67.1^a^	76,7±2,5^bc^	151.9±78.9^ab^	77,1±6,3^a^
Najeh	219.6±124.1^a^	73,4±3,9^a^	185.3±128.1^b^	76±10,3^a^
XAR-VF00.13-1-2-1-2-1	218.2±119.7^a^	74,1±5,5^ab^	169.2±113.7^b^	76±7,7^a^
XBJ90.04-2-3-1-1-1-2A	164.2±63.4^a^	78,2±2,6^c^	129.4±73.1^b^	76±11,4^a^

Values followed by the same letter per column are not significantly different at p = 0.05 (Duncan test).

**Fig 3:**
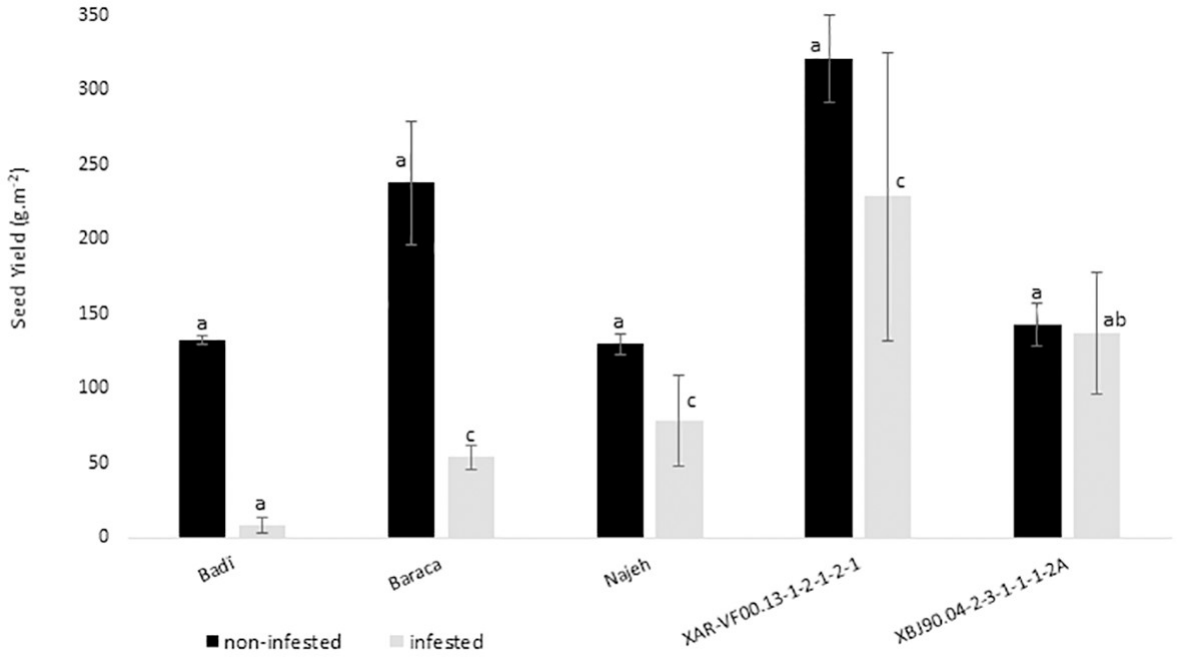


*Orobanche* parasitism effect on the host plant development resulted in significant seed yield losses for all the studied genotypes (Fig 3). Compared to free *Orobanche* plants, seed yield decreases varied from 3.9% recorded for XBJ90.04-2-3-1-1-1-2A to 93.9% observed for the susceptible check Badi. Respective decreases of 77.4%, 39.5% and 28.8% were observed for Baraca, Najeh and XAR-VF00.13-1-2-1-2-1. Among all the tested genotypes, XAR-VF00.13-1-2-1-2-1 was the most productive under *O*. *foetida* infested conditions with 228.4 g.m^-2^ representing 3 and 4 times the seed yield recorded for the resistant checks Najeh (78.3 g.m^-2^) and Baraca (53.8 g.m^-2^). For cv. Baraca which is reported to be resistant to *O*. *crenata*, SY varied from 237.8 g.m^-2^ to 53.8 g.m^-2^ (77.4% less) under free and *Orobanche* infested fields, respectively.

#### Chlorophyll content index and chlorophyll fluorescence

**Fig 4:**
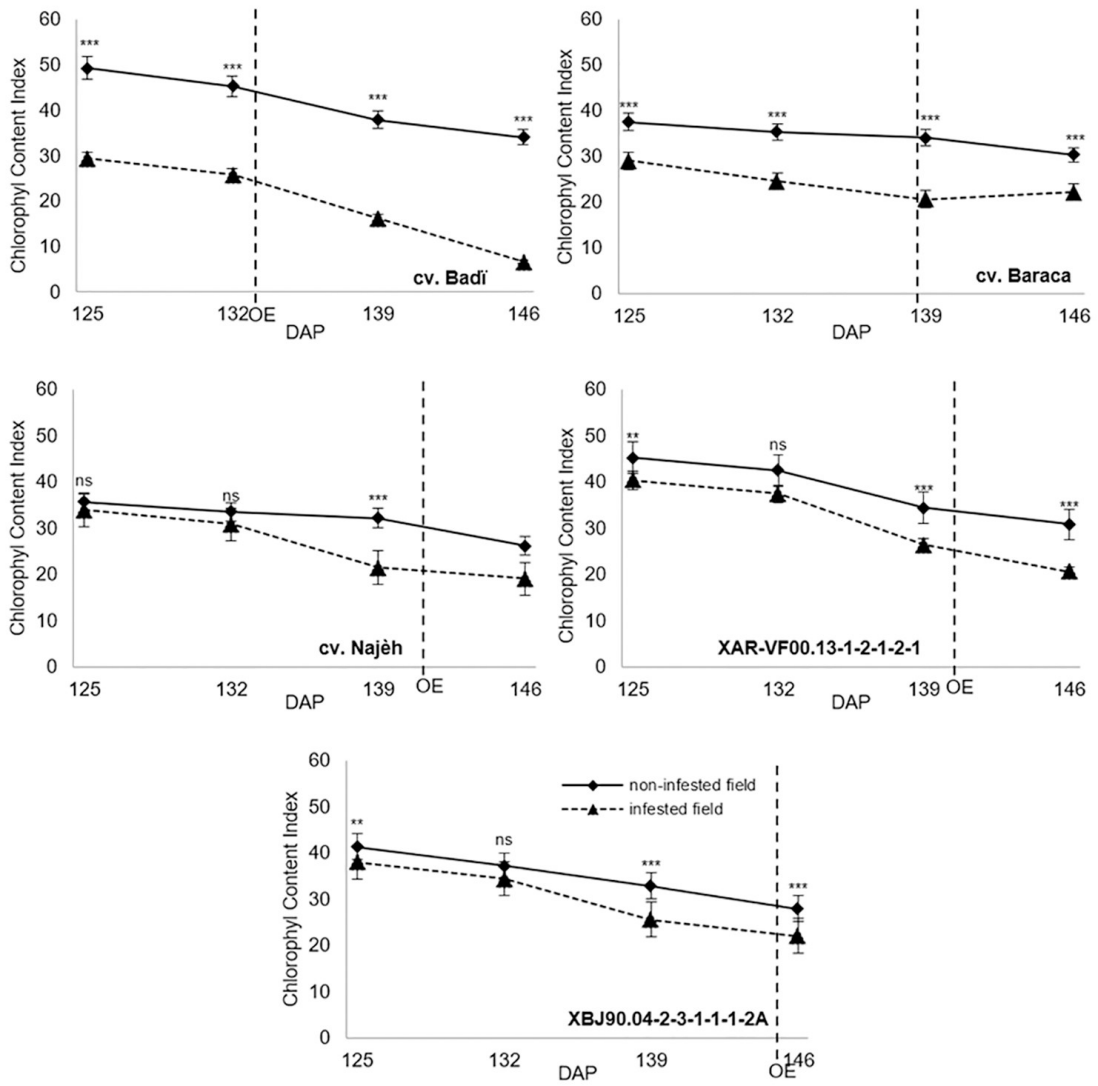


**Fig 5:**
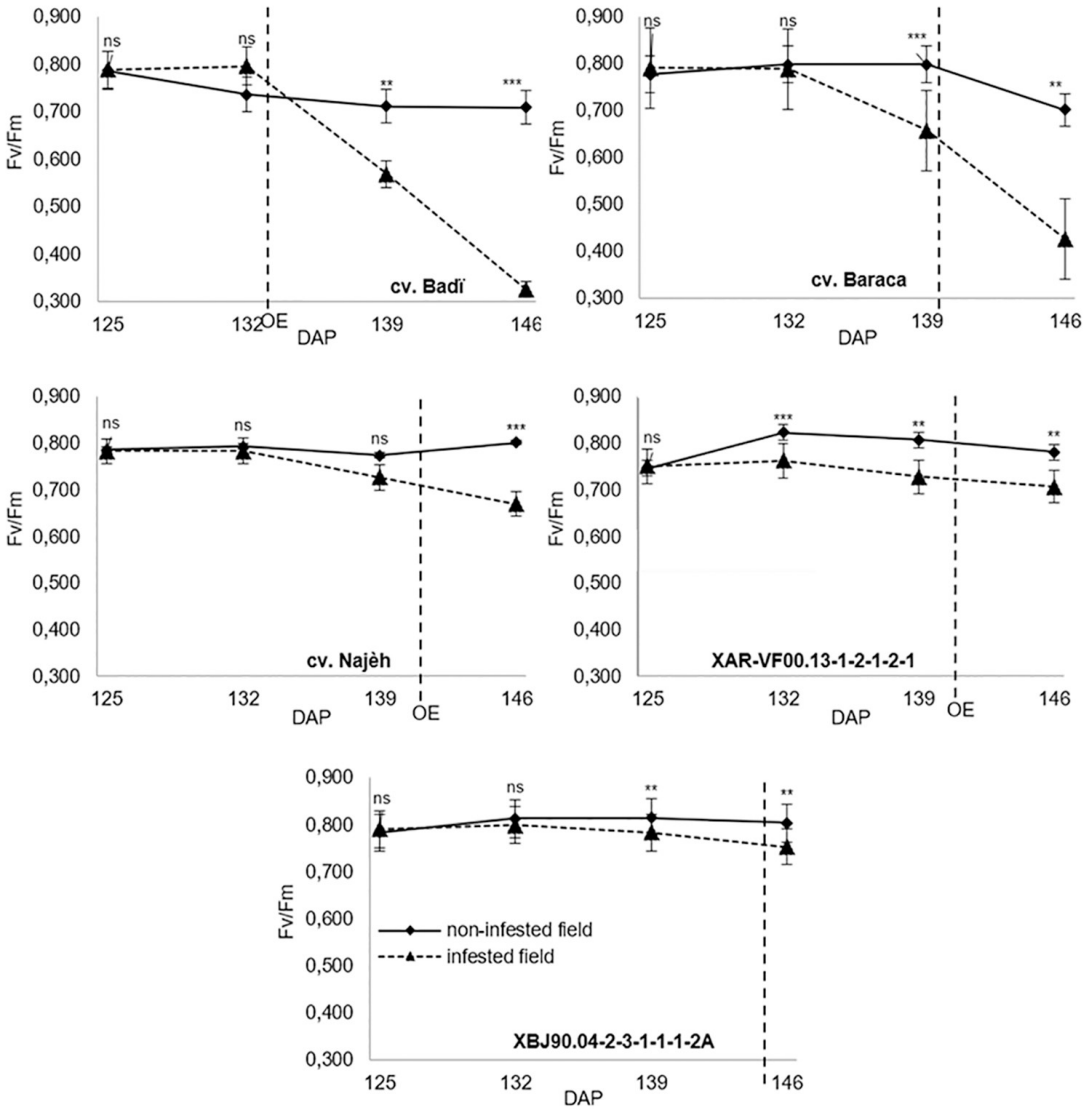


*Orobanche* parasitism significantly affected (*P* ≤ 0.01) the host plant chlorophyll content index (CCI) and F_v_/F_m_ ratio for all the studied genotypes (Figs 4 and 5). All genotypes showed significant difference between infected and non-infected plants before *Orobanche* emergence. Between 125 and 146 DAP, CCI decreases under *O*. *foetida* infestation varied from 23.6% for Baraca to 77.2% recorded for Badi. Respective decreases of 19.4% and 30.8% were recorded for the same genotypes under free *Orobanche* field. Clear differences were observed for the CCI between infected and free *Orobanche* plants. Such differences were more pronounced for the susceptible check Badi. Variation between 125 and 146 DAP, varied from 4.2% for Baraca to 46.4% observed for Badi.

F_v_/F_m_ ratio was significantly affected by *Orobanche* parasitism (Fig 5). In infected plants F_v_/F_m_ decreased by 58.8% (0.789 to 0.325) for the susceptible check Badi against only 9.9% (0.787 to 0.709) observed for free *Orobanche* plants. Decreases of 46.2%, 14.5%, 5.9% and 4.7% were recorded, respectively, for Baraca, Najeh, XAR-VF00.13-1-2-1-2-1 and XBJ90.04-2-3-1-1-1-2A. Except for the susceptible check Badi, all other genotypes showed significant differences in F_v_/F_m_ between infected and free *Orobanche* plants before *Orobanche* emergence (Fig 5). High decreases of F_v_/F_m_ ratio were observed for Badi and Baraca after 139 DAP against only slight decreases recorded for Najeh and both selected advanced lines XAR-VF00.13-1-2-1-2-1 and XBJ90.04-2-3-1-1-1-2A. At 146 DAP, comparison of F_v_/F_m_ ratio between infected and free *Orobanche* plants showed differences of 54.2% (0.709 vs 0.325), 39.3% (0.702 vs 0.426) and 16.5% (0.802 vs 0.670) recorded for Badi, Baraca and Najeh, respectively, against only 6.4% (0.804 vs 0.753) for XBJ90.04-2-3-1-1-1-2A and 9.5% (0.781 vs 0.707) for XAR-VF00.13-1-2-1-2-1.

## Discussion

Results from the first-year screening showed high variability for the resistance to *O*. *foetida* in the tested collection. Resistance to broomrapes is not only the capacity of the genotype to limit the parasite development and the damages that causes, but also the capacity of that same genotype to grow and produce grains under such parasitism attack. Two advanced lines, XBJ90.04-2-3-1-1-1-2A and XAR-VF00.13-1-2-1-2-1 were identified and selected for their high resistance level and good SY under high *O*. *foetida* infestion. Both genotypes showed a low infestation level compared to resistant and susceptible checks. Previous studies showed that *Orobanche* causes early wilting symptoms in parasitized plants and results in a shortening of the reproductive phase and affecting significantly the flowering, pod setting, plant biomass and seed production [13, 16, 28]. Such effects were highly pronounced for cv. Badi, for which *O*. *foetida* has severely restrained plant growth, and resulted in almost complete damage and yield losses. Moderate effect of the parasite on plant development and seed production was observed for both selected advanced lines XAR-VF00.13-1-2-1-2-1 and XBJ90.04-2-3-1-1-1-2A. Results also showed that despite the biomass decreases recorded for different studied genotypes, no significant effect of *Orobanche* parasitism was observed on the host plant’s WC. Indeed, due to the parasitic burden and resources sinking the host plant has limited its biomass and dry matter production and allocation in order to keep its physiological functioning through a normal and optimum water content. It was also reported that *Orobanche* parasitism effects on host plant growth and biomass production and allocation are directly associated with the infestation level [2, 9, 10]. Ennami et al., [29] reported a high negative correlation between both faba bean and lentil plant’s growth and *O*. *crenata* development attributing that to the competition between the host and the parasite for nutrients. Other previous studies reported that the detrimental effect of both *O*. *foetida* and *O*. *crenata* on faba bean grain yield can reach up to 90–100% depending on the infestation severity and the broomrape-crop association [30, 31]. In addition, our results showed significant differences in CCI between infected and noninfected plants for the five tested genotypes, even before *Orobanche* emergence. These results indicate that this parameter could be very useful for early detection of the underground *Orobanche* infestation. In fact, several studies reported the importance of the number of host leaves and their greenness in plant eco-physiological studies because they provide information about physiological responses of plants under stress conditions [32, 33]. Decreases in CCI that were observed in parasitized plants could be explained by the parasite nutritional requirements that limits the normal growth and functioning of the host plant. Similar results were reported for tomato/*P*. *ramosa* pathotype [34, 35]. Shamsullah et al. [35], found that, compared to noninfected plants, *P*. *ramosa* decreased chlorophyll content in tomato leaves by 29.17%. Shen et al., [23] reported also similar results with *Mikania micrantha*/*Cuscuta campestris* and showed, also, that despite the CCI decrease observed on the *M*. *micrantha* leaves, there was no significant effects of *C*. *campestris* parasitism on chlorophyll a:b ratio. In our study, CCI decreases were associated with photosynthetic characteristics variation in the host plant leaves. *O*. *foetida* affected the photosynthetic system through significant decreases of the leaves CCI and F_v_/F_m_ ratio which was increasingly pronounced over time, especially for the susceptible check Badi. For all the genotypes, *O*. *foetida* parasitism significant decreased F_v_/F_m_ ratio after 146 DAP. compared to non-infected plants. Before *Orobanche* emergence, only resistant genotypes showed significant difference in F_v_/F_m_ between infected and free *Orobanche* plant. Despite *Orobanche* parasitism effect, the two genotypes XAR-VF00.13-1-2-1-2-1 and XBJ90.04-2-3-1-1-1-2A were able to maintain a normal functioning of their PSII to the same level as the free-*Orobanche* plants even after *Orobanche* emergence. This was not the case for the susceptible check Badi. These results along with the chlorophyll fluorescence analyses indicated that F_v_/F_m_ could be used not only for the quantification of stress caused by *Orobanche* parasitism and early detection of the underground infestation but also the screening and identification of high resistant genotypes. Similar results were reported by Mauromicale et al. [34] who showed that F_v_/F_m_, which is proportional to the PS II quantum yield and well correlated with the photosynthesis quantum yield [36], was significantly decreased by *P*. *ramosa* attack on tomato plants. In the same study, the authors demonstrated that the F_v_/F_m_ reduction is mainly induced by an effect on the variable fluorescence (F_v_) resulting in PS II electron transport damage. Similarly, Rousseau et al. [18], reported that F_v_/F_m_ in *Arabidopsis thaliana* leaves was impacted by *P*. *ramosa*. Also, other studies showed that *C*. *reflexa* induced a sink-dependent stimulation of net photosynthesis on *Ricinus communis* [37] and that such infestation by *C*. *campestris* decreases host stomatal conductance, transpiration, chlorophyll content, and soluble protein concentration on *M*. *micrantha* [23]. These results are contrasting with other studies [38, 39] who reported that broomrape affects host biomass and yield and related traits with only minor disturbance in host’s leaves tissue but no perceptible effects on photosynthetic rate. More recently, Ennami et al. [29] showed that effective quantum yield of open photosystem II, (Fm’-F)/Fm’, was significantly reduced by *O*. *crenata* attack on susceptible faba bean and lentil genotypes. The effects caused cause by the parasitic weeds on the different parameters may directly or/and indirectly affect the functioning of the photosynthetic system and rate and therefore affect the growth of the host plant.

## Conclusions

*O*. *foetida* can affect faba bean host plants in/through different ways and at a big range of scales, from the root to the leaves through the whole plant. Out of the initially larger tested faba bean collection, the two genotypes XAR-VF00.13-1-2-1-2-1 and XBJ90.04-2-3-1-1-1-2A expressed the highest resistance level to *O*. *foetida* and showed a moderate and limited effects of the parasite on plant development and seed production. For both genotypes, the high resistant level was reflected by limited effects of the parasite on plant growth, biomass production, seed yield and physiological functioning of the host plants. Significant variations in CCI and F_v_/F_m_ were observed from individual plants between the tested genotypes and between infected and noninfected plants. The significant positive correlation observed between CCI, F_v_/F_m_ and high resistance level to *Orobanche* may suggest the integration of these physiological traits in plant selection and screening for resistance to broomrapes. These practical screening tools could be coupled with other new smart imaging technologies for early detection of the root parasitic weeds infestation.
